# Impaired Osteoblast Differentiation in Annexin A2- and -A5-Deficient Cells

**DOI:** 10.1371/journal.pone.0107482

**Published:** 2014-09-15

**Authors:** Damian C. Genetos, Alice Wong, Thomas J. Weber, Norman J. Karin, Clare E. Yellowley

**Affiliations:** 1 Department of Anatomy, Physiology and Cell Biology, School of Veterinary Medicine, University of California Davis, Davis, CA, United States of America; 2 Systems Toxicology, Pacific Northwest National Laboratory, Richland, WA, United States of America; Georgia Regents University, United States of America

## Abstract

Annexins are a class of calcium-binding proteins with diverse functions in the regulation of lipid rafts, inflammation, fibrinolysis, transcriptional programming and ion transport. Within bone, they are well-characterized as components of mineralizing matrix vesicles, although little else is known as to their function during osteogenesis. We employed shRNA to generate annexin A2 (AnxA2)- or annexin A5 (AnxA5)-knockdown pre-osteoblasts, and determined whether proliferation or osteogenic differentiation was altered in knockdown cells, compared to pSiren (Si) controls. We report that DNA content, a marker of proliferation, was significantly reduced in both AnxA2 and AnxA5 knockdown cells. Alkaline phosphatase expression and activity were also suppressed in AnxA2- or AnxA5-knockdown after 14 days of culture. The pattern of osteogenic gene expression was altered in knockdown cells, with *Col1a1* expressed more rapidly in knock-down cells, compared to pSiren. In contrast, *Runx2*, *Ibsp*, and *Bglap* all revealed decreased expression after 14 days of culture. In both AnxA2- and AnxA5-knockdown, interleukin-induced STAT6 signaling was markedly attenuated compared to pSiren controls. These data suggest that AnxA2 and AnxA5 can influence bone formation *via* regulation of osteoprogenitor proliferation, differentiation, and responsiveness to cytokines in addition to their well-studied function in matrix vesicles.

## Introduction

Annexins comprise a class of calcium-dependent, phospholipid-binding proteins that are broadly expressed in eukaryotic cells. They are predominately localized within the cell, where they mediate such cellular processes as exocytosis and endocytosis, membrane structure and generation of lipid rafts, formation or regulation of ion channels, and cytokinesis. A subset of annexins have extracellular roles, and participate in regulation of inflammation, coagulation and fibrinolysis (reviewed in [1]). More recently, they have been identified as key mediators in maintaining endothelial and hematopoietic stem cells within the bone marrow niche [2], [3] and as pivotal regulators of metastasis and adhesion of prostate cancer cells within bone [4].

Of the 12 Annexins expressed in mammals, Annexins A1, A2, A4, A5, A6 and A7 are expressed within cells of the chondrogenic and osteoblastic lineage [5]–[7]. To date, their function within these cells has primarily focused upon a putative role in matrix mineralization. AnxA5 is involved in endochondral ossification, and is sequentially expressed during vasculogenesis and formation of the cartilage anlage [8], [9]. During embryogenesis and post-natal skeletal development, AnxA2 and AnxA5 are present in matrix vesicles secreted by hypertrophic chondrocytes and osteoblasts [10]–[15]. Similarly, Annexins A1, A4, and A7 are also found within matrix vesicles from mineralizing osteoblasts [16]. However, little data exist as to whether, and when, AnxA2 or AnxA5 exert cell-autonomous roles in an osteoblast. We have reported that AnxA5 is involved in transducing a biophysical signal–fluid shear stress–into increases in intracellular calcium and inducing gene transcription in osteoblasts [17]. With regards to the hematopoietic component of the skeleton, exogenous AnxA2 increases the formation of human bone marrow multinucleated cells, TRAP-positive staining, and dentine resorption [18]. Certain of these effects occur indirectly, as AnxA2 increases pre-osteoclast proliferation by increasing GM-CSF production from bone marrow stromal cells and activated T cells [19], and promotes ERK1/2-dependent RANKL secretion from bone marrow stromal cells [17], [20], [21]. Gillette and Nielsen-Preiss demonstrated that over-expression of AnxA2 in human osteosarcoma cells facilitates the terminal stages of osteogenic differentiation, specifically matrix mineralization [22], although if AnxA2 exerted a role prior to mineralization was not examined.

While these data indicate a role for AnxA2 in matrix mineralization, whether either AnxA2 or AnxA5 have cell-autonomous effects on processes occurring prior to mineralization–proliferation and osteogenic differentiation–remains unexamined. In this study, we examined the influence of depletion of *AnxA2* or *AnxA5* (AnxA2kd and AnxA5kd, respectively) upon the proliferation and osteogenic differentiation of the pre-osteoblast MC3T3-E1 cell line. Reduced expression of AnxA2 or AnxA5 decreased proliferation and altered the dynamic course of osteogenic differentiation compared to pSiren (Si) control cells. Mechanistically, AnxA2kd and AnxA5kd each demonstrated decreased responsiveness to the anti-inflammatory cytokine interleukin 4 (IL-4), indicating that both AnxA2 and AnxA5 are required for maximal responsiveness. In total, these data demonstrate cell-autonomous roles for both AnxA2 and AnxA5 in proliferation of pre-osteoblasts, matrix maturation and mineralization.

## Results

### Annexin A2 and A5 expression in knockdown cell lines

**Figure 1:**
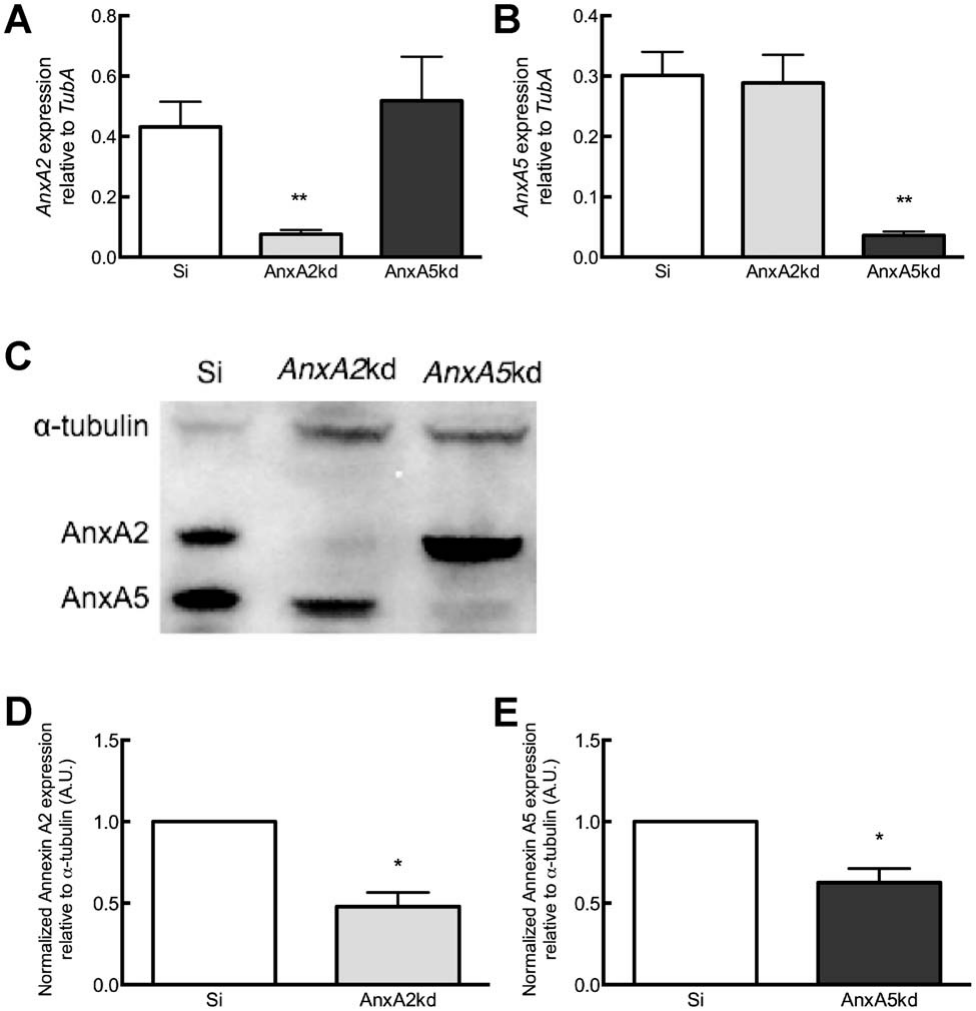
Characterization of Annexin levels in knockdown cells. (A) qPCR analysis of *AnxA2* expression in pSiren control cells (Si) and cells stably transfected with either AnxA2 or AnxA5 shRNA (*AnxA2*kd and *AnxA5*kd cells respectively). (B) qPCR analysis of *AnxA5* expression in cells stably transfected with either AnxA2 or AnxA5 shRNA. Each bar represents mean transcript normalized to α-tubulin ± SEM, n = 3–5. **represents statistically significant difference from Si, p<0.01. (C) Representative western immunoblot for AnxA2, AnxA5 and α-tubulin protein expression in Si, *AnxA2*kd and *AnxA5*kd cells. (D) Quantitation of AnxA2 protein levels in Si and *AnxA*2kd cells. (E) Quantitation of AnxA5 protein levels in Si and *AnxA5*kd cells. Data are first normalized to α-tubulin and then to Si control. Each bar represents mean ± SEM, n = 6. *represents statistically significant difference from Si control, p<0.05.

Stable MC3T3-E1 cell lines deficient in *AnxA2* and *AnxA5* expression were generated as described in Materials and Methods. There was a significant reduction (>80%) in *AnxA2* mRNA expression in *AnxA2*kd cells compared to Si-transfected controls (**Figure 1A**), and there was no compensatory change in *AnxA5* m*RNA* in *AnxA2*kd cells. Similarly, there was a significant reduction in *AnxA5* mRNA in *AnxA5*kd cells compared to Si control, and no effect of *AnxA5* depletion upon *AnxA2* mRNA expression (**Figure 1B**). Changes in Annexin expression were also confirmed at the protein level by western immunoblot (**Figure 1C**). Densitometric analysis relative to α-tubulin showed that AnxA2 protein expression in *AnxA2*kd was approximately 50% of Si control (**Figure 1D**). AnxA5 protein expression was reduced by approximately 40% in *AnxA5*kd cells compared to Si control (**Figure 1E**).

### Proliferation decreases and the dynamics of osteogenic differentiation are disrupted in Annexin-deficient cells

**Figure 2:**
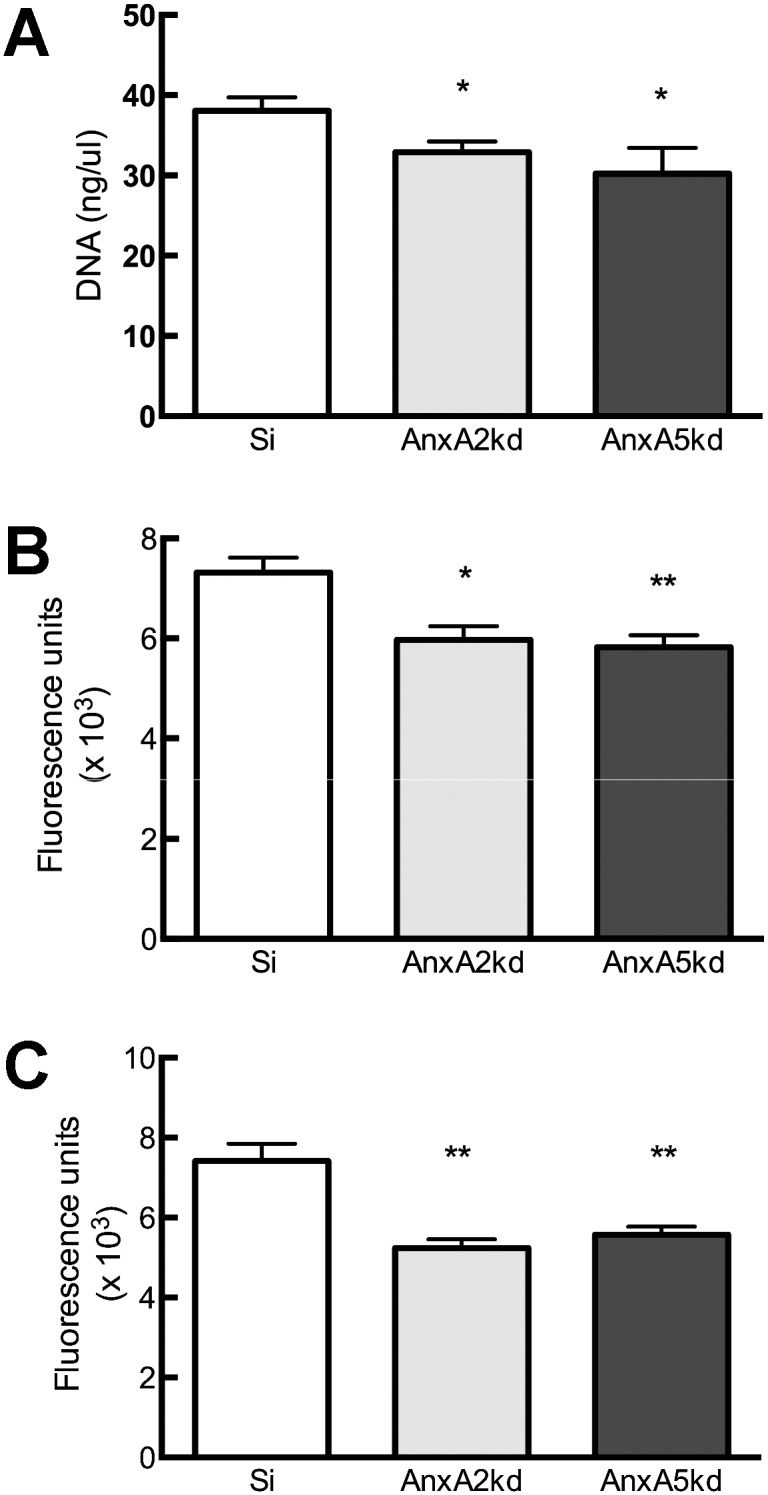
Decreased proliferation in *AnxA2*kd and *AnxA5*kd cells. (A) Quantification of DNA concentration 24 hrs after cell seeding. Bars represent mean DNA concentration (ng/µL sample)±SEM, n = 5–8. (B) Quantification of Calcein-AM fluorescence 24 hrs after cell seeding. Bars represent mean calcein fluorescence units ± SEM, n = 3–8. (C) Quantification of Alamar blue absorbance 48 hrs after cell seeding. Bars represent mean optical density ± SEM, n = 8. *, **represent statistically significant difference from Si, *p<0.05, **p<0.01.

Having demonstrated significant reduction of AnxA2 and AnxA5 mRNA and protein, we next examined whether alterations in AnxA2 or AnxA5 expression influenced pre-osteoblast proliferation. Total DNA content was significantly reduced in *AnxA2*kd (14% reduction) and in *AnxA5*kd (20% reduction) cells, relative to Si control (**Figure 2A**). Similar reductions in proliferation were observed using Calcein-AM (reductions of 29% and 30%; **Figure 2B**) and Alamar Blue (18% and 20%; **Figure 2C**) assays in *AnxA2*kd and AnxA5kd, respectively.

**Figure 3:**
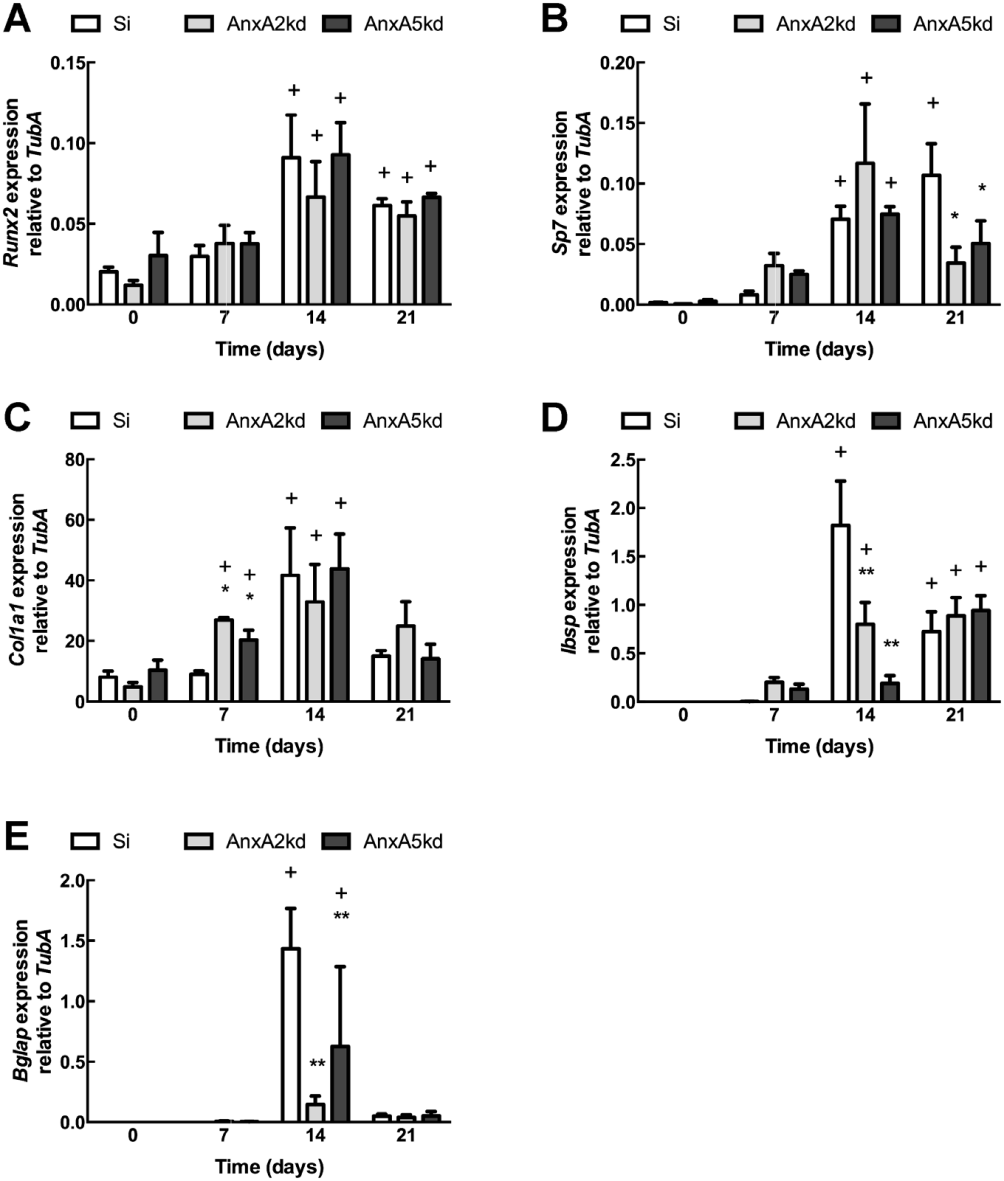
Effects of *AnxA2* and *AnxA5* knockdown on expression of genes associated with osteogenic differentiation. qPCR analysis of (A) *Runx2*, (B) *Sp7*, (C) *Col1a1*, (D) *Ibsp,* and (E) *Bglap* expression in undifferentiated Si, *AnxA2*kd and *Anx5*kd cells (day 0) and in cells cultured in differentiation medium for 7, 14 and 21 days. Each bar represents mean transcript normalized to α-tubulin ± SEM, n = 3. *, **, ***represent statistically significant difference from Si at same time point, *p<0.05, **p<0.01, ***p<0.001; + represents statistically significant difference from same genotype on day 0, p<0.05.

Annexins are implicated in matrix mineralization by nature of their presence in matrix vesicles isolated from osteoblasts and chondroblasts, although there is no data whether Annexins may have a role in earlier stages of osteogenic differentiations, specifically matrix formation or maturation. Thus, we examined the influence of AnxA2 or AnxA5 reduction upon markers of osteogenic differentiation. Si control cells showed a dynamic pattern of gene expression associated with osteogenic differentiation (*Col1a1*, *Runx2*, *Ibsp*, *Sp7*, *Spp1* and *Bglap*) [23]. For Si controls, initiation of osteogenesis at 0 days resulted in significant increases in the osteogenic transcription factors *Runx2* and Osterix (*Sp7*) after 14 days in culture, and was maintained at 21 days of culture compared to day 0 controls (**Figures 3A and B**). A similar time course of *Runx2* induction and level of expression was observed in both *AnxA2*kd and *AnxA5*kd cells (**Figure 3A**), although *Sp7* expression decreased significantly at 21 days in culture compared to Si controls (**Figure 3B**). For each cell type, osteogenic differentiation increased *Col1a1* expression, although induction expression occurred earlier in *AnxA2*kd and *AnxA5*kd cells compared to Si controls (**Figure 3C**), suggesting that the ordered process of osteogenic differentiation was subtly altered in *AnxA2*kd and *AnxA5*kd cells. Non-collagenous proteins implicated in matrix maturation and ordered deposition of hydroxyapatite–bone sialoprotein (*Ibsp*) and osteocalcin (*Bglap* or *Ocn*)–each revealed altered patterns of expression in knockdown cells compared to Si controls. *Ibsp* expression was maximally expressed after 14 days in culture in Si; *AnxA2*kd cells demonstrated a statistically significant increase in *Ibsp* also at 14 days, although expression was significantly lower compared to Si, and *Ibsp* expression in *AnxA5*kd cells was not different from 0 days (**Figure 3D**). Similarly, *Bglap*/*Ocn* expression was attenuated in *AnxA2*kd and *AnxA5*kd cells compared to Si controls after 14 days in culture (**Figure 3E**).

**Figure 4:**
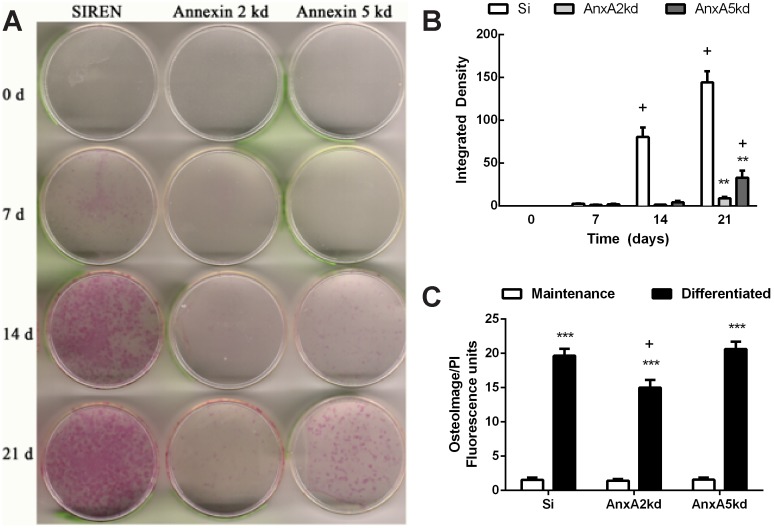
Effects of AnxA2 and AnxA5 knockdown on ALP and hydroxyapatite. (A) ALP activity staining in MC3T3-E1 cells (MC3T3), Si, *AnxA2*kd and *AnxA5*kd cells after culture in osteogenic differentiation media for 7, 14 and 21 days, representative images from n = 3 biological replicates. (B) Quantitation of ALP staining intensity. Bars represent mean integrated signal intensity ± SEM, n = 3. + represents statistically significant difference from same genotype on day 0, p<0.05. **represented statistically significant different from Si at the same day, p<0.01. (C) OsteoImage staining for hydroxyapatite in Si, *AnxA2*kd and *Anx5*kd cells cultured with differentiation media for 5 weeks. Each bar represents OsteoImage fluorescence normalized to PI ± SEM, n = 3. ***represents statistically significant difference from maintenance media within the same genotype, ***p<0.001; + represents statistically different from Si in same media composition, p<0.01.

Osteogenic differentiation was monitored also by histochemical staining for alkaline phosphatase, an early marker of osteogenesis, and mineral deposition into the extracellular matrix. Si control cells demonstrated a progressive increase in staining intensity with time in culture that was apparent visually (**Figure 4A**) and quantitatively (**Figure 4B**). In contrast, for both *AnxA2*kd and *AnxA5*kd cells, there was a marked reduction in staining intensity with time in culture. *AnxA2*kd cells demonstrated only slight punctate staining after 21 days in culture. *AnxA5*kd cells similarly demonstrated less staining compared to Si controls, although staining was more pronounced in *AnxA5*kd compared to *AnxA2*kd at both days 14 and 21, indicating that *AnxA5*kd cells have an osteogenic potential that is intermediate between Si controls and *AnxA2*kd cells. All cell lines deposited significant quantities of mineral as determined by OsteoImage mineralization assays at 5 weeks, although *Anx2*kd cells deposited significantly less mineral than both Si and *Anx5*kd cells (**Figure 4C**).

### AnxA2 and AnxA5 are dynamically-expressed during osteogenic differentiation

**Figure 5:**
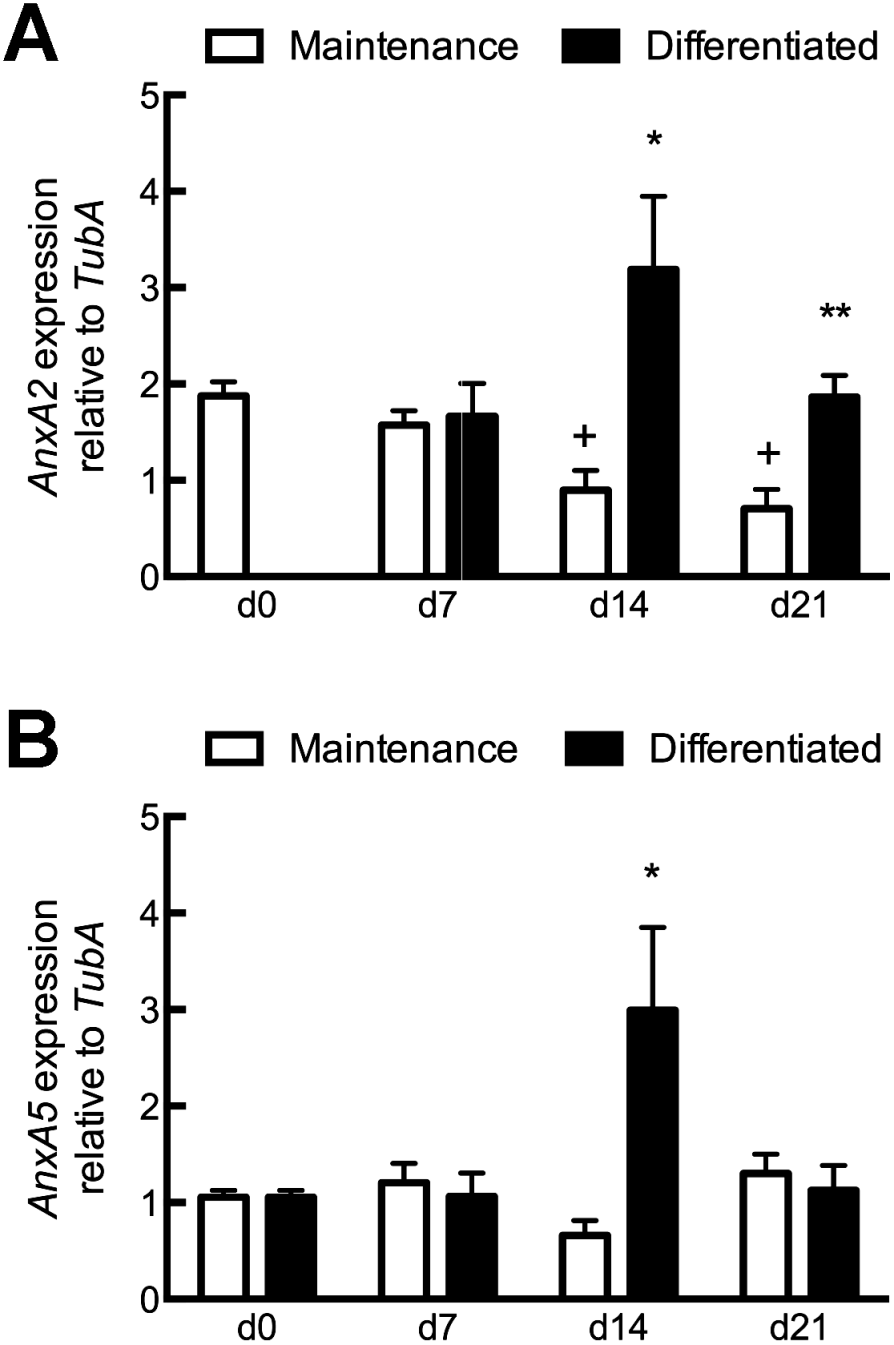
Effects of osteogenic differentiation on expression of AnxA2 and AnxA5. qPCR analysis of (A) *AnxA2* or (B) *AnxA5* expression in undifferentiated Si cells at day 0 and in cells cultured in maintenance or osteogenic media 7, 14 and 21 days. Each bar represents mean transcript normalized to α-tubulin ± SEM, n = 3–5. *, **represent statistically significant difference from maintenance control at same time point, *p<0.05, **p<0.01; + represents statistically different from same media composition on day 0, p<0.05.

Expression of *AnxA2* and *AnxA5* was monitored in MC3T3 cells cultured in the absence (maintenance media) and presence of ascorbic acid and β-glycerophosphate (osteogenic), reagents traditionally used to induce osteogenic differentiation. In maintenance media, *AnxA2* expression demonstrated reduction in expression with increased culture time that reached statistical significance after 14 or 21 days in culture (**Figure 5A**, white bars); similarly, there was no effect of time in culture on *AnxA5* expression (**Figure 5B**, white bars). In contrast, both *AnxA2* and *AnxA5* expression increased as a function of time when cells were cultured in osteogenic media, with *AnxA2* demonstrating significantly increased expression at d14 and d21, and *AnxA5* revealing maximal expression at d14 but then returning toward baseline at d21. These data indicate that *AnxA2* and *AnxA5* expression as a function of time in culture under osteogenic differentiation-inducing conditions are dynamic.

### Reductions in *AnxA2* or *AnxA5* attenuate IL-4-induced signaling

**Figure 6:**
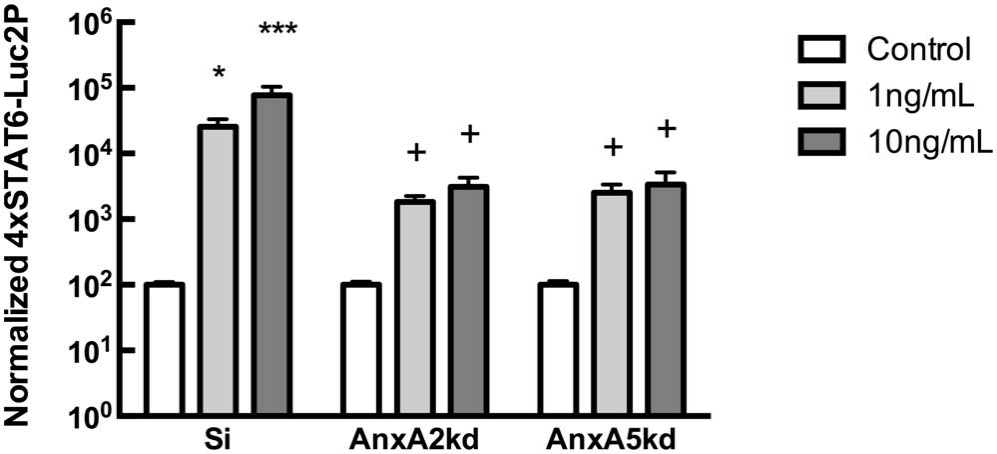
IL-4-induced STAT6 signaling in parental and mutant cells. Si, AnxA2*kd*, or AnxA5*kd* cells were transiently transfected with p4xSTAT6-Luc2P and treated with 0, 1, or 10 ng/mL IL-4 for 24 hours, after which Luciferase activity was measured. Each bar represents mean Luciferase activity ± SEM normalized to vehicle control, n = 3. *, ***represent statistically significant difference from Si control, *p<0.05, **p<0.01; + represents statistically significant difference compared to Siren at same IL-4 concentration, p<0.001.

Having demonstrated that reducing *AnxA2* or *AnxA5* attenuate pre-osteoblast proliferation and alter the timing of osteogenic differentiation, we began to examine mechanistic explanations for the observed results. It has been reported that AnxA2 associates with signal transducer and activator of transcription 6 (STAT6) and stimulates STAT6 transcriptional activity in prostate cancer cells [24]. To examine whether reduced *AnxA2* or *AnxA5* expression influences STAT6 signaling in osteoblastic cells, Si, *AnxA2*kd or *AnxA5*kd cells were transiently transfected with p4xSTAT6-Luc2P, a plasmid encoding firefly luciferase driven by four copies of the STAT6 DNA binding site. Cells were subsequently challenged with 0, 1, or 10 ng/mL IL-4. Si-transfected cells demonstrated a dose-dependent increase in Luciferase activity in response to IL-4 (**Figure 6**). In contrast, neither *AnxA2*kd nor *AnxA5*kd cells demonstrated significant increases in luciferase activity in response to IL-4, indicating that reduced AnxA2 or AnxA5 expression compromises STAT6 signaling. There were no significant differences in luciferase activity between genotypes in the absence of IL-4.

## Discussion

Annexins comprise a class of calcium-binding proteins with a diverse array of intracellular and extracellular functions, including matrix mineralization in hypertrophic cartilage and bone. Both AnxA2 and AnxA5 are present in matrix vesicles isolated from both chondrocytes and osteoblasts, where they are thought to act as membrane channels to allow Ca^2+^ influx and hydroxyapatite crystal formation. Over-expression of AnxA2 correlates with increased alkaline phosphatase activity and calcium deposition in osteoblast cultures [22]. However, there are inherent problems associated with protein overexpression, such as mis-trafficking of proteins, that could potentially confound the results and conclusions [25]. Further, previous work examined the terminal stage of osteogenic differentiation–mineralization–without examining whether other stages, such as proliferation and extracellular matrix deposition and maturation, and are unable to indicate if AnxA2 only affects matrix deposition, or multiple stages of osteogenic differentiation. Thus, we sought whether AnxA2, and AnxA5, have cell-autonomous roles during multiple stages of osteogenic differentiation–proliferation, matrix formation and organization, and matrix mineralization [23].

Pre-osteoblastic MC3T3-E1 cells expressed AnxA2 and AnxA5 at similar levels, as determined by both mRNA and protein (**Figure 1A–C**). shRNA techniques stably decreased *AnxA2* or *AnxA5* transcript levels by 82% or 88%, respectively, compared to Si-transfected controls (**Figures 1A and 1B**), which decreased AnxA2 or A5 protein expression by 52% or 37%, respectively (**Figures 1C–E**); reductions in AnxA2 or AnxA5 elicited no compensatory change in expression of AnxA2 or A5 at the mRNA or protein level, consistent with work by Belluoccio *et al.*, who found no change in *AnxA2* or other annexins in compound *AnxA5^−/−^;AnxA6^−/−^* mice [8]. Both AnxA2kd and AnxA5kd cells have decreased proliferative capacity compared to Si cells (**Figures 2A–C**), and suppression of AnxA2 by shRNA similarly decreases proliferation of adenocarcinoma [26], breast cancer [27], and multiple myeloma [28] cells.

Osteogenic differentiation occurs with the serial induction of *Runx2* followed by Osterix (*Sp7*); in all cell types examined, the pattern of *Runx2* expression was similar, suggesting that the influence of *AnxA2* or *AnxA5* reductions upon osteogenic differentiation either is *Runx2*-independent or involves processes initiated after induction of *Runx2*. In contrast, *Sp7* was only transiently expressed in *AnxA2*kd and *AnxA5*kd cultures compared to Si, wherein its expression was significantly lower in either knockdown cell type at 21 days of culture (**Figure 3B**); these suggest that both *AnxA2* and *AnxA5* are required for maximal induction of *Sp7* expression under the course of osteogenic differentiation. Attenuated expression of *Ibsp* and *Bglap*, genes associated with matrix maturation and proper hydroxyapatite deposition, was also observed in mutant cells, and the temporal pattern of *Col1a1* expression in *AnxA2*kd and *AnxA5*kd cells was disrupted compared to Si controls. Osteogenic differentiation was also impaired when examining ALP activity (**Figures 4A and 4B**) and calcium deposition (**Figure 4C**). These data indicate that normal expression of *AnxA2* and *AnxA5* may be required for the ordered, sequential processes inherent in osteoblast differentiation.

We observed that *AnxA2* and *AnxA5* are each dynamically expressed during the course of osteogenic differentiation. Both *AnxA2* and *AnxA5* expression increased significantly after 14 days in culture (**Figures 5A and 5B**); *AnxA2* remained elevated, whereas *AnxA5* returned to baseline, at 21 days. The observed changes in osteogenic gene expression (**Figures 3A–E**) occur at the same time that *AnxA2* and *AnxA5* are maximally expressed. This suggests a causative link between *AnxA2* or *AnxA5* expression and osteogenesis, which is confirmed by our differentiation studies in *AnxA2*kd and *AnxA5*kd cells.

*AnxA2*kd cells do not phenocopy *AnxA5*kd cells. While knockdown of either AnxA2 or AnxA5 decreased DNA content to the same degree (**Figure 2A–C**), there exist subtle differences in the effect of *AnxA2* or *AnxA5* knockdown upon osteoprogenitor differentiation. Reducing expression of either Annexin altered gene expression, but the effects were generally more pronounced in *AnxA2*kd cells compared to *AnxA5*kd cells. For example, AnxA5kd cells still demonstrated positive staining for ALP activity after 21 days of culture, whereas positive staining was nearly absent in *AnxA2*kd cells (**Figure 4A**). This was also reflected in calcium deposition into the extracellular matrix: after 5 weeks of culture, there was significantly less extracellular calcium in AnxA2kd cells compared to AnxA5kd, which themselves showed no difference compared to Si (**Figure 4C**). Despite attenuated ALP staining and expression of *Ibsp* and *Bglap*, total calcium deposition was not affected in *AnxA5*kd cells compared to Si controls. Because Ibsp and Bglap are involved in matrix organization, it is possible that the approaches we used to not fully demonstrate differences in matrix composition between Si and *AnxA5*kd cells; further analysis by FT-IR for mineral-matrix ratio, scanning electron microscopy, or atomic force microscopy are necessary in order to do so. Nonetheless, data suggest that AnxA2 and AnxA5 likely exert non-redundant roles in osteogenesis.

Mechanistically, the observed results could involve AnxA2 or AnxA5 functioning as ion channels to regulate cytosolic calcium levels, critical determinants of progression through the cell cycle and gene transcription [29], [30]. Alternately, Annexins may regulate gene transcription directly and indirectly. Annexin A4 enhances NF-κB subunit p50 transcriptional activity [31], [32], and Annexin A1 expression positively correlates with NF-κB activity in breast cancer metastasis [33], [34]. In prostate cancer cells, AnxA2 physically interacts with STAT6 to stabilize cytosolic levels of phosphorylated STAT6 and promote its nuclear localization [24]. Transfection of cells with a STAT6-reporter plasmid demonstrated that IL-4-induced signaling is attenuated in *AnxA2*kd or *AnxA5*kd cells compared to Si controls (**Figure 6**). T helper lymphocytes (Th2 cells) secrete IL-4, -5, -10, and -13, which are generally anti-inflammatory by inhibiting the activity of cytokines secreted from Th1 cells, such as IL-1, -2, -6, and -12 [35]. IL-4 stimulates proliferation and mineralization by enhancing hydroxyproline accumulation, calcium deposition, and osteocalcin secretion [36], [37]; because we observed attenuated response to IL-4 and osteogenic differentiation in *AnxA2*kd and *AnxA5*kd cells, it is possible that AnxA2 and AnxA5 function as regulators of transcription in osteoblastic cells. Indeed, Belluoccio *et al.* reported significant alteration in the transcriptome of compound *AnxA5^−/−^;AnxA6^−/−^*, wherein 56% of differentially-regulated genes (defined by 3-fold difference compared to control) were related to cell survival, involving cell motility, apoptosis, cell cycle, cell proliferation and differentiation and tumor suppressor/survival [8].

Direct regulation of osteogenesis may also involve the capacity of AnxA2 to bind and sequester Sclerostin, an inhibitor of the Wnt signaling pathway [38]. Manipulation of Wnt signaling through altered expression of antagonists or the Wnt co-receptor Lrp5 exerts robust effects upon bone formation and homeostasis [39]–[41], and biophysical factors that influence Sclerostin expression in mature osteoblasts also regulate *AnxA2* expression [42], [43], although whether this is correlative or causative requires further study. Nonetheless, we have provided the first evidence that AnxA2 and AnxA5 are involved in pre-osteoblast proliferation, the timing and magnitude of osteogenic gene expression, matrix maturation, and responsiveness to anti-inflammatory cytokines. These results provide the foundation to consider novel multifaceted roles for *AnxA2* and *AnxA5* in osteogenesis.

## Materials and Methods

### Cell culture

MC3T3-E1 (clone 14; ATCC) osteoblastic cells were stably-transfected with pSIREN vector (Si; Clontech) with or without siRNA targeting AnxA2 or AnxA5. Generation of AnxA2- and AnxA5-knockdown cells is described below. Cells were maintained under 95% ambient air, 5% CO_2_ in humidified incubators. Medium was Minimal Essential Medium, alpha modification (Invitrogen), supplemented with 10% FBS (Invitrogen) and 1% penicillin-streptomycin. Puromycin (2 µg/ml) was added to the media to maintain selection of the cells. Cells were routinely sub-cultured with 0.05% trypsin and except where noted, cells were seeded at a density of 3,000/cm^2^, and studies performed the following day. For osteogenic differentiation studies growth medium was supplemented with 50 µg/mL ascorbic acid (Sigma) and 5 mM β-glycerophosphate (Sigma).

### Generation of AnxA2 and AnxA5 knockdown cells

AnxA2 knockdown cells (*AnxA2*kd) were generated as previously reported [44]. AnxA5 knockdown cells (*AnxA5*kd) were created using the pSIREN vector (Clontech) containing *AnxA5* shRNA (target sequence from Qiagen siRNA-SI00899101). shRNA was ligated into pSIREN, transformed into DH5-alpha competent cells (Invitrogen), and amplified in Luria broth containing ampicillin (50 µg/mL). Before transfection, the plasmids were sequenced to confirm the presence of the shRNA (data not shown). Plasmids (2–4 µg) were transfected into MC3T3-E1 cells using Clonfectin (Clontech) and three days later the cells were placed in media containing puromycin (2 µg/ml) for selection. After one week, surviving colonies were placed into 48-well plates and maintained in culture. Three weeks later, cells were tested for AnxA5 expression by western immunoblot.

### Western immunoblot analysis

Cells were washed with phosphate buffered saline (PBS) and whole cell protein lysates were collected in RIPA buffer (15 mM Tris-HCl, pH 7.6, 1% Igepal CA-630, 0.5% sodium deoxycholate, and 0.1% SDS), supplemented with a protease inhibitor cocktail (Pierce-ThermoFisher). Samples were separated on 10% Bis-Tris gels (Invitrogen), transferred onto 0.2 µM nitrocellulose membranes, and blocked in 5% non-fat milk in Tris-buffered saline. Proteins were detected with antibodies directed against AnxA2 (1∶1000, Santa Cruz), Anx A5 (1∶1000, Santa Cruz), and α-tubulin (1∶1000, Cell Signaling). A secondary goat anti-rabbit antibody linked to horseradish peroxidase (1∶1000, Jackson ImmunoResearch) was used before developing in ECL (Invitrogen).

### Quantitative PCR

One µg of total RNA was reverse-transcribed with Superscript II Reverse Transcription Kit (Invitrogen) and qtPCR was performed using primer and TaqMan probe sets (Applied Biosystems, Foster City, CA) and QuantiFast Probe PCR kit (Qiagen, Valencia, CA) on a Mastercycler realplex2 (Eppendorf, Westbury, NY). Amplification conditions were 95°C for 3 min, followed by 40 cycles at 95°C for 3 s and 60°C for 30 s. Taqman Primer and probe sets *AnxA2*, *AnxA5, Col1a1, Ibsp, Runx2, Sp7*, *Bglap*, and *TubA* were purchased from Applied Biosystems. Quantitative PCR results were normalized to *TubA* transcript level for MC3T3-E1 cells to Si controls to obtain 2^−ΔΔCt^ [45].

### ALP activity staining and quantitation

Cells were seeded in 35-mm plates and grown for 7, 14 and 21 days in osteogenic differentiation medium and stained with an ALP activity staining kit (Sigma) according to manufacturer’s instructions. Briefly, cells were washed with pre-warmed PBS (37°C) and fixed with citrate-acetone-formaldehyde solution. After fixing, cells were washed with filtered de-ionized water, incubated with alkaline dye mixture for 15 min, washed and then allowed to air-dry. Digital images were acquired using a flatbed scanner. Integrated density was calculated as the area of the image multiplied by the mean gray value, when white was defined as 0 and black as 255.

### Proliferation assays

Cells were seeded at 5,000 cells/well in 96-well plates and 3 different proliferation assays were performed: 1) 25 hrs after seeding, cells were washed with PBS, lysed with water, and a NanoDrop (Thermo-Fisher) was used to measure DNA concentration; 2) Calcein-AM (2 µg/ml; Invitrogen) was added to the cells in Earle’s Balanced Saline Solution 24 hrs after seeding, incubated for 30 min at 37°C, then fluorescence was read at 485/530 nm (Biotek); 3) Alamar blue (Invitrogen) was added directly to the cells 24 hrs after seeding. Cells were maintained in culture for a further 24 hrs after which absorbance was read at 570 nm.

### OsteoImage Mineralization Assay

Cells were in 96-well plates, and osteogenic differentiation media was added the following day. After 5 weeks in differentiation media, the cells were washed with PBS, fixed with MeOH and incubated with fluorescent OsteoImage staining reagent, which binds to hydroxyapatite, *per* the manufacturer’s protocol (Lonza Walkersville, Inc.). Fluorescence was read at 485/530 nm for OsteoImage staining. Propidium iodide (5 µM) was added for 60 min and the plate was quantitated at 530/620 nm. OsteoImage values were normalized to propidium iodide values.

### STAT6 signaling assay

Si, *AnxA2*kd or *AnxA5*kd cells were seeded at a density of 20,000 cells/well in 48-well plates, and the next day were and transfected with a cocktail composed of (per well) 1.2 µL X-tremeGENE HP (Roche Applied Science), 250 ng 4xSTAT6-Luc2P (plasmid 35554, kindly provided by Dr. Axel Nohturfft through Addgene), and 100 ng of pRL-TK (Promega). Twenty-four hours later, cells were treated with 0, 1, or 10 ng/mL IL-4 (PeproTech) in growth medium; after 24 hours of treatment, wells were washed in PBS, scraped and collected in passive-lysis buffer, and analyzed using Dual-Luciferase Reporter System (Promega) and TD-20/20 luminometer (Turner Systems).

### Statistical analysis

One- or two-way ANOVA was performed on proliferation, PCR, western blot quantitation and OsteoImage data. Individual significance was determined by Tukey’s multiple comparison test. Statistical significance was considered at p<0.05 for all tests performed.
